# A Case of Squamous Cell Carcinoma of the Nasal Cavity Treated With Total Rhinectomy

**DOI:** 10.7759/cureus.23576

**Published:** 2022-03-28

**Authors:** Theresa A Schneider, Oscar Gryn, Matthew Lutz

**Affiliations:** 1 Microbiology and Immunology, Des Moines University, Des Moines, USA; 2 Otolaryngology - Head and Neck Surgery, Western Reserve Hospital, Cuyahoga Falls, USA

**Keywords:** free flap reconstruction, nose reconstruction, nasal cavity mass, rhinectomy, squamous cell carcinoma (scc)

## Abstract

Squamous cell carcinoma of the nasal cavity is a relatively rare cancer. Five-year recurrence-free survival rates have a large range, which may be due to the small patient population available to study. Recurrence rates vary based on the treatment regimen and aggressiveness of the surgical approach. Total rhinectomy is not often performed due to its invasive nature and extensiveness of reconstruction required afterward. This report will cover a patient who presented with squamous cell carcinoma of the left nasal vestibule and was treated with total rhinectomy and radiation therapy.

## Introduction

Squamous cell carcinoma (SCC) comprises about 50% of sinonasal malignancies [1]. Only approximately 3% of head and neck SCCs occur in the nasal cavity [2]. Risk factors for SCC of the nasal cavity include wood dust, leatherworking, and cigarette smoke [3]. The average age of patients with SCC of the nasal cavity is 65.8 years [4]. Studies on SCC of the nasal cavity have varied in the predominant sex of affected patients, although it appears that men tend to be reported at a higher rate in comparison to women [1,2,4,5].

Often, the first symptoms of SCCs of the nasal vestibule are epistaxis and nasal obstruction with possible signs of ulceration to the affected area. Later symptoms include contour changes to the nose. Due to the nonspecific nature of their symptoms, SCCs of the nasal cavity are often diagnosed months after symptoms begin [6]. Treatment for SCC of the nasal cavity is typically done with surgery to excise the primary lesion along with reconstruction using autologous material or a nasal prosthesis [6].

## Case presentation

**Figure 1:**
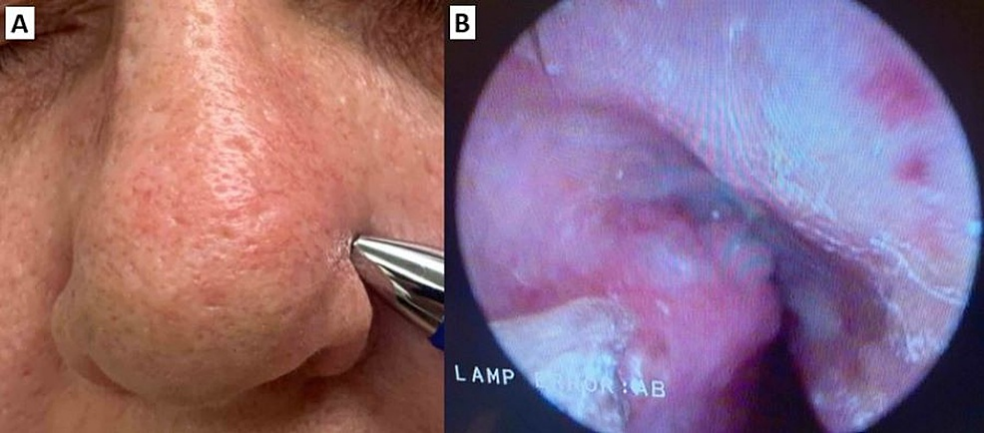
Initial examination of left nasal mass. Distortion of nasal contour (A). Rigid nasal endoscopic examination revealed left nasal cavity with an ulcerated lesion involving left superior nasal septum and left superior nasal vestibule (B).

A 58-year-old female with poorly controlled diabetes mellitus (DM) and a 60 pack-year smoking history presents to otolaryngology with a chief complaint of a left-sided nasal mass for six weeks. Prior treatment with two rounds of antibiotics did not lead to any improvement. Physical examination at the initial visit was notable for septum deviated to the right as well as crusting and scabbing over the superior aspect of the left nasal septum and left superior nasal vestibule extending to the outer wall of the nasal cavity (Figure 1A). No active bleeding or palpable cervical or supraclavicular lymphadenopathy was noted at that time. 

**Figure 2:**
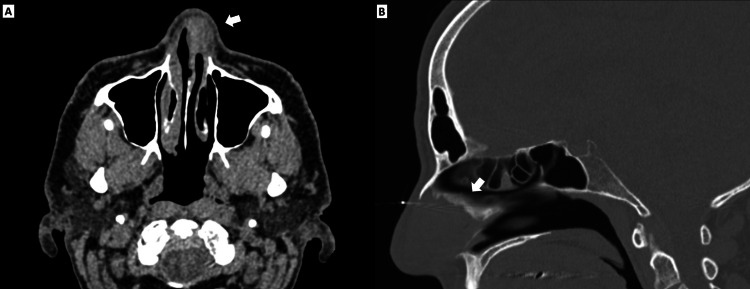
Contrast-enhanced transverse (A) and sagittal CT sections (B). Maxillofacial CT scan noting mass in left anterior nasal vestibule measuring 1.5 × 1.6 × 1.4 cm.

A rigid nasal endoscopic examination (Figure 1B) revealed an ulcerated lesion involving the left superior nasal septum and left superior nasal vestibule. A maxillofacial CT scan noted a mass filling the left anterior nasal vestibule measuring 1.5 × 1.6 × 1.4 cm (Figures 2A, 2B). A positron emission tomography (PET) scan revealed increased uptake in the left nasal cavity and left neck near the submandibular lymph nodes, which was concerning for cervical metastasis. 

**Figure 3:**
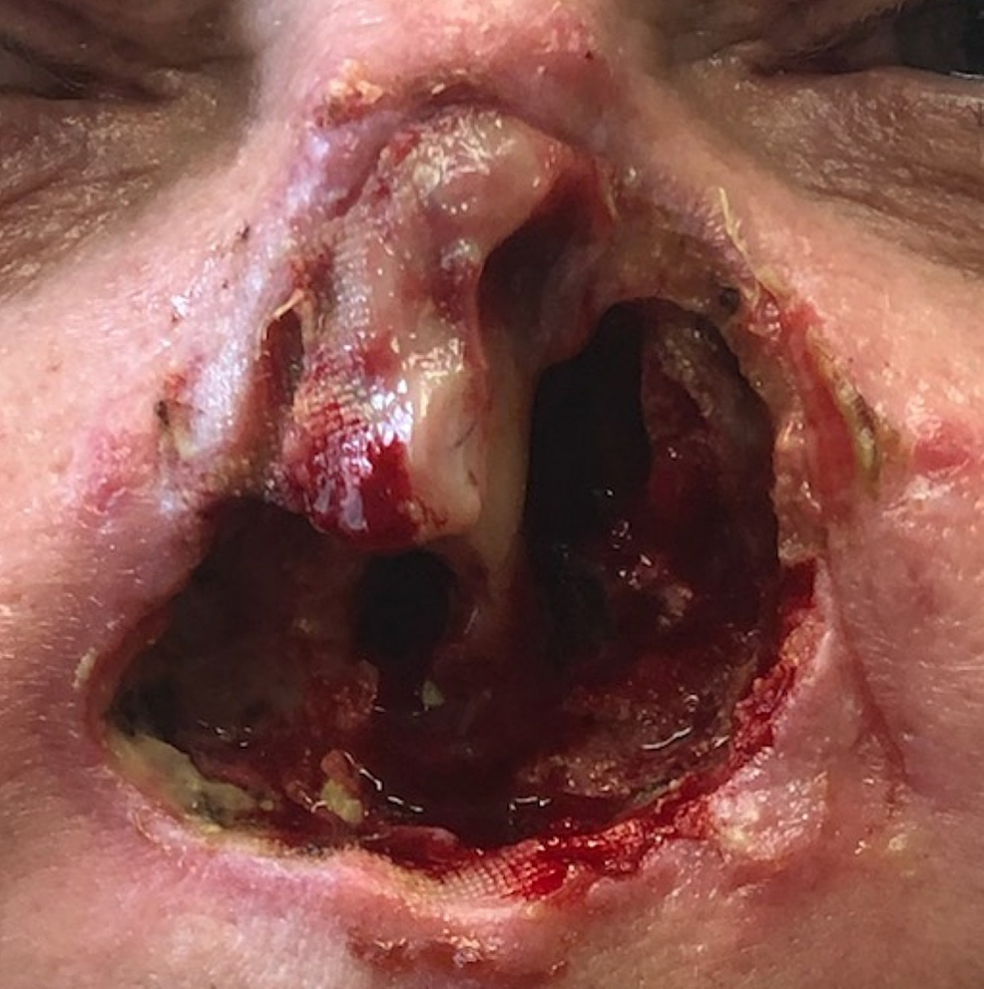
Total rhinectomy postoperative result.

Three months after initial presentation, the patent underwent a scheduled partial rhinectomy. After frozen section analysis was performed which showed residual SCC on the anterior margin, it was determined that a total rhinectomy would be required (Figure 3). Final pathology determined staging to be T4aN1M0 and the final size of the tumor was at least 3.2 cm. There was evidence of perineural invasion requiring referral for radiation therapy. ­­

## Discussion

This case presents a combinational approach to treating SCC of the nasal cavity including surgical resection of the mass with adjuvant radiation. Less than 1% of head and neck cancers are SCCs of the nasal vestibule, with SCC being the most common histology [2,6]. Early stages of SCC of the nasal cavity are often treated with radiotherapy, as well as surgical excision in some cases. Later stages require surgical excision with radiation therapy depending on the depth of invasion. Research has shown that surgical excision with clear margins is the standard of care and results in the lowest rate of recurrence. This is often done by an external approach, including lateral rhinotomy, partial rhinectomy, and total rhinectomy [6]. Patients who have insufficient margins are at significantly increased risk of local or regional recurrence of disease, even if radiation is given afterward [7]. Total rhinectomy is often deferred with a preference for radiation therapy for multiple reasons. Patients may be concerned about the cosmetic appearance and emotional impact of such an extensive surgery. Physicians may be weary of such a radical surgery because of concerns with reconstruction. Reconstruction can be done using a prosthesis or through surgical repair which involves reconstituting each of the three layers of nasal tissue: the inner lining, the structural support of bone and cartilage, and the external envelope of skin. The inner lining can be reconstructed using local flaps, grafts, mucosal flaps, or free flaps. Structural support may be grafted from calvarium, septal cartilage and bone, rib, auricular cartilage, or a combination of these grafts. Finally, the external envelope is often repaired using a paramedian forehead graft or free flap with donor sites involving the radial forearm, skin, and ear flaps along superficial temporal vessels, or skin along the dorsal metacarpal artery [7]. Nonetheless, aggressive surgical resection is key, and its omission can lead to the risk of recurrence both locally and regionally [8]. These factors influenced our decision to use an aggressive surgical approach to allow for the lowest risk of recurrence and highest cure rate. 

There were several challenges to total rhinectomy in this patient. This patient has poorly controlled DM, which will likely prolong wound healing. In patients with DM, trauma can result in chronic wounds due to delayed wound healing. An open wound can allow for infection to occur, and increased glucose levels provide an environment for bacteria to thrive [9]. Proper blood glucose control is critical in our patient to allow for adequate wound healing to occur. The patient also has a significant smoking history. Smoking is known to damage microcirculation resulting in limited oxygenation to tissue and leading to poor wound healing [10].

The nose is an anatomically complex structure and our patient will face many obstacles with reconstruction. Often a nasal prosthesis is used in partial or total rhinectomies to improve final cosmetic and functional results [11]. However, due to financial reasons, our patient declined reconstruction using a prosthesis, which leaves autologous and microvascular grafts. Also, our patient will be undergoing radiation therapy due to evidence of perineural invasion. Radiation often results in fibrosis and decreased vascularity in the affected area. Skin can become firm and secondary ulcers or fistulas can form [12]. Further studies may investigate reconstruction efforts after total rhinectomy using techniques that do not involve skin flaps but still result in adequate function. There may also be a benefit in researching reconstruction efforts after radiation therapy.

## Conclusions

Here we report on a case of SCC of the nasal cavity requiring total rhinectomy and radiation therapy in a patient with several comorbidities including tobacco abuse and poorly controlled DM. Current treatment options for SCC of the nasal cavity include radiation therapy and surgical excision. This case demonstrates that total rhinectomy is a viable surgical approach to provide resection of SCC of the nasal cavity.
